# Radiological and Imaging Evidence in the Diagnosis and Management of Microbial Infections: An Update

**DOI:** 10.7759/cureus.48756

**Published:** 2023-11-13

**Authors:** Aditya Vaishnav, Gurukiran Gurukiran, Osazuwa Ighodaro, Venkataramana Kandi

**Affiliations:** 1 Cardiothoracic Surgery, St Bartholomew, London, GBR; 2 Orthopaedics and Trauma, Royal Shrewsbury Hospital, Shrewsbury, GBR; 3 Emergency Department, NHS, Basildon, GBR; 4 Clinical Microbiology, Prathima Institute of Medical Sciences, Karimnagar, IND

**Keywords:** management of infectious diseases, radiological investigations, culture, microscopy, diagnosis, self-limiting, microbial infections

## Abstract

Microbial infections are extremely prevalent throughout the world. Bacteria, fungi, parasites, and viruses generally cause them. Most microbial infections spread from humans to humans and from animals to humans. A vast majority of microbial infections are self-limiting. However, some microbial infections result in severe morbidity and mortality. The diagnosis of microbial infections generally depends on the direct demonstration of microbes in human clinical specimens through microscopy followed by culture. Some microbes are uncultivable, and among those that are cultivable, some take a very long time to grow in the laboratory. This causes delays in the diagnosis that may result in poor patient outcomes. Serological and molecular methods like enzyme-linked immunosorbent assay (ELISA) and polymerase chain reaction (PCR), respectively, have been extensively used to diagnose infectious diseases. However, these require costly infrastructure and adequate personnel training. In this context, alternative, more efficient, and rapid detection methods for the diagnosis of microbial infections are warranted. In this review, we comprehensively discuss the role played by radiological investigations in the diagnosis and management of infectious diseases.

## Introduction and background

Microbial infections are among the most common ones affecting the health of humans as well as animals [1]. Microbes, including bacteria, fungi, parasites, and viruses, cause infections that range from mild and self-limiting to severely debilitating diseases that cause death and result in severe morbidity among the affected population [2]. Most microbial infections can be successfully treated with antimicrobial therapeutic agents. However, some microbial infections caused by Dengue virus, Chikungunya virus, Zika virus, Ebola virus, and others are difficult to treat due to the unavailability of a specific therapeutic agent [3]. Additionally, some microbial infections like tuberculosis, pneumonia, measles, polio, and others are preventable with timely vaccinations [4]. The cause for concern with microbial infections is the possibility of these infections being transmitted to other people, thereby resulting in the spread of infections covering vast geographical regions and population groups, resulting in epidemics (spread to different regions in the same country) and pandemics (spread to different countries in the world and affecting a large number of people) [5]. Besides, microbial infections could result in the long-term presence of the causative microbe in different organs of humans without being noticed for years [2]. The diagnosis of microbial infections can be performed by various methods that include microscopy, culture, serology, and molecular methods like polymerase chain reaction (PCR) and matrix-associated laser desorption ionization-time of flight-mass spectroscopy (MALDI-ToF-MS), among others [6].

**Figure 1:**
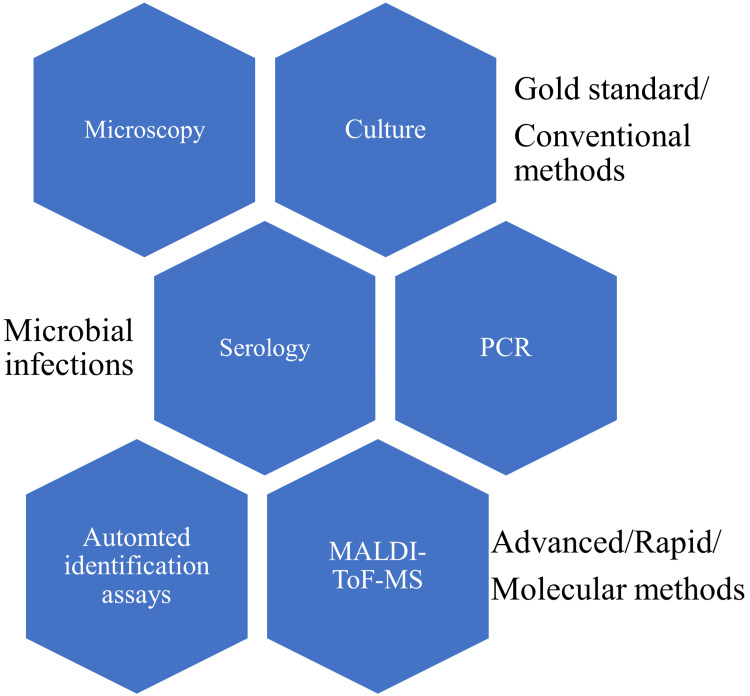
Diagnostic methods used in the diagnosis of microbial infections PCR: polymerase chain reaction; MALDI-ToF-MS: matrix-associated laser desorption ionization-time of flight-mass spectroscopy

Diagnosis by culture is considered the gold standard for the diagnosis of common bacterial infections. However, several bacterial infections can also be diagnosed by using serological methods like enzyme-linked immunosorbent assays (ELISA). A majority of fungal and parasitic infections are diagnosed using microscopic methods like potassium hydroxide (KOH) mount and stool wet mount, respectively. Despite the availability of culture techniques for the diagnosis of fungal infections, they are less reliable due to the slow growth of fungi, which could delay the treatment. Many viral infections are diagnosed using serological and molecular methods like ELISA and PCR, respectively. Viral culture methods, although well established, require special infrastructure like biological safety cabinets (BSC) and personal protective equipment (PPE). Moreover, viral culture establishments/laboratories could become a source of spread of deadly/lethal viruses among people within and outside the laboratory. Different available methods to diagnose microbial infections are shown in Figure 1.

In this review, we explore the utility of radiological and other imaging techniques in diagnosing various microbial infectious diseases.

## Review

Radiological evidence in the diagnosis of infectious diseases generally implies the use of X-rays, computerized X-rays (CT), radiofrequency (magnetic resonance imaging/MRI), and sound waves (ultrasonography) to diagnose infections. These imaging techniques are applied to identify the anatomical structure and abnormalities during infection and/or inflammation [7]. Radiological evidence in the diagnosis and management of microbial infections appears to be crucial with reference to infections caused by some microbes, like the novel Severe Acute Respiratory Syndrome Coronavirus-2 (SARS-CoV-2) and Mycobacterium tuberculosis, among others. The major drawback with the application of imaging techniques appears to be the indirect evidence of infection rather than the identification of the causative agent. Moreover, radiological evidence could also assist in the diagnosis and prognosis of microbial infections, thereby providing an efficient means for patient management. Microbe-associated internal organ infections and inflammations that result in abscesses and fluid accumulations can be visualized using imaging techniques like ultrasonography and computed tomography (CT). Imaging techniques, although not helpful in the specific diagnosis and identification of the causative microbes, are less harmful and noninvasive [8]. Additionally, imaging techniques allow physicians and surgeons to accurately identify the location of infections, thereby assisting in the collection of specimens. Radiolabeling of specific microbes, including bacteria and viruses, was previously used to identify the infection caused by Escherichia coli and the extent of viral spread, respectively [9].

In a recent study, 99mTc-labelled, colistin-encapsulated, theranostic liposomes were successfully used to identify and treat Pseudomonas aeruginosa infection [10]. This study's results emphasize the potential role of imaging techniques in the diagnosis and treatment of microbial infections. Another recent prospective study assessed the role of CT imaging in the differential identification of an infected area at a sterile body site. The results of this study revealed that CT imaging features demonstrating attenuation >10 Hounsfield units (HU), entrapped gas, and wall enhancement could indicate the presence of microbial infection [11]. Tc-leukocyte scintigraphy was applied to a patient with recurrent spikes of fever. It was observed that the patient was infected in the lungs by SARS-CoV-2, as evidenced by the increased uptake of Tc-leukocytes in both lungs noticed through a CT chest. This imaging observation was correlated with a positive PCR test for SARS-CoV-2 infection [12].

The major drawback of radiological imaging is that this technique cannot differentiate between microbial infection and sterile inflammation [13]. Imaging techniques have sparse utility in the diagnosis of infections caused by viruses. This may be attributed to no or fewer fluid accumulations during such infections, in contrast to bacterial and mycobacterial infections, where there is evidence of fluid collection that can be visualized in radio imaging. Radiological evidence may prove beneficial in the diagnosis of fever of unknown origin, endocarditis, pneumonia, and other clinical conditions that have atypical and complex patient presentations [14].

Various imaging techniques like bioluminescence (BLI) and fluorescence imaging [fluorescence reflectance imaging (FRI)] and fluorescence-mediated tomography (FMT) and nuclear medicine methods like positron emission tomography (PET), single photon emission computed tomography (SPECT), and magnetic resonance imaging (MRI) were assessed for their utility in the diagnosis of microbial infections. It has been noticed that high-resolution and increased contrast imaging of the MRI may assist in the diagnosis of soft tissue infections caused by microbes, including bacteria and viruses [15].

Dual imaging utilizing PET/CT, PET/MRI, and SPECT/CT was suggested to improve the diagnosis of infectious diseases. Additionally, it was noticed that a simple USG may be helpful in the identification of infection foci in the emergency/casualty departments as a point-of-care test. However, it was observed that most imaging methods cannot identify an early infection where there is no change in the anatomical structure and the absence of other presenting features like fluid accumulation and organ/tissue/cell changes [16].

According to the consensus statement regarding the diagnosis of peripheral bone infections (PBI) in adults released by the European Bone and Joint Infection Society (EBJIS), the European Society of Microbiology and Infectious Diseases (ESMID), the European Society of Radiology (ESR), and the European Association of Nuclear Medicine (EANM), there are pros and cons to the diagnosis of PBI that apply different radiological imaging techniques. The consensus proposes that ultrasound is efficient in the diagnosis of soft tissue infections and biopsies, easily available, low in cost, and has no radiation burden. However, USG was noted to be less useful in the diagnosis of bone infections. The consensus suggests that CT is helpful in performing image-guided biopsies and is easily available at an affordable cost. However, the drawbacks of CT include low diagnostic accuracy and a moderate radiation burden. MRI was suggested as the most preferred method in the diagnosis of PBI, with similar advantages as CT, but was noted to present with false positivity. Additionally, the consensus proposes that nuclear medicine techniques like PET-CT and SPECT are more accurate and highly sensitive in the diagnosis of PBI [17].

Bacterial infections and imaging

Bacteria account for the majority of infections among humans, mostly attributed to low immune responses and antimicrobial resistance. The application of radiological imaging fails to differentiate bacterial infections from other microbial infections and non-infectious etiologies. Targeted imaging, wherein a contrast agent is conjugated with a specific molecule that targets the bacterial agent, was suggested as an improved technique. Radioisotope-labeled antibodies like human polyclonal immunoglobulin (99 mTech-netium), Mycobacterium bovis (BCG)-specific antibody (131Iodine), and Pseudomonas aeruginosa-specific antibody (125Iodine) were found to be efficient in the diagnosis of bacterial infections. Radiolabelled antibiotics, antimicrobial proteins/peptides, metabolites/ligands, bacteriophages, and nucleic acid degradation materials were also suggested in the diagnosis of bacterial infections [18].

Animal models of microbial keratitis were analyzed using a multicolor fluorescence imaging device (FluoroPi). FluoroPi utilizes fluorescent optical reporters/SmartProbes to differentially identify Gram-positive and Gram-negative bacterial infections. The limit of detection using this procedure was identified as 103 to 104 colony-forming units (CFU) per milliliter (mL) of sample [19].

Animal experiments on lung infection models have suggested that variations in the L- and D-amino acids were useful in predicting bacterial infections through imaging [20]. Polymyxin B (PMB) is an old antibiotic that is least used in the treatment of bacterial infections due to its neurotoxicity and nephrotoxicity. PMB is increasingly used to treat infections caused by Gram-negative bacteria that are multi-drug resistant (MDR) as a last resort antibiotic [21].

Considering the fact that imaging techniques fail to distinguish between sterile fluid accumulations and those caused by bacterial infections, maltodextrin-based probes have been utilized to specifically identify bacteria-mediated fluid accumulations. Maltodextrin is a glucose source for bacterial growth and metabolism [22,23].

Additionally, antibiotic-labeled probes have been attempted to identify specific bacterial infections caused by Gram-positive [NIR fluorophore IRDye 800 CW conjugated vancomycin (vanco-800CW)] and Gram-negative bacteria [radiolabeling PMB with 99 mTc via a succinimidyl-6-hydrazinonicotinate hydrochloride (HYNIC) linker] [24].

Bacteria infect bones following orthopedic trauma that results in fractures. Fracture-related infections (FRIs) are extremely common throughout the world. However, a majority of such infections remained undiagnosed until the bacteria spread to different parts of the body and resulted in sepsis. Therefore, early diagnosis of FRIs is extremely essential to minimize the morbidity among such patients. Bacteria-targeted fluorescence imaging was recently explored to identify bacterial colonization on implant devices. The implant devices were incubated with a near-infrared fluorescent tracer composed of the antibiotic vancomycin and the fluorophore IRDye800CW (vanco-800CW). Later, imaging was performed on the devices, which demonstrated fluorescence that correlated with bacterial cultures. This study result emphasized the role of imaging in the diagnosis of FRIs [25].

Synthetic amyloid peptides, also called peptins, were used as vector probes that could adhere to microbial and mammalian proteins. PET scans utilizing these radiolabeled probes, including [68Ga]Ga-NODAGA-PEG2-P2, that target Escherichia coli, have been found to efficiently identify intracellular targets [26].

The nitroreductase (NTR) activity demonstrated by bacteria has been utilized to differentiate between cancer-related fluid accumulations/inflammation from bacterial infections. The near-infrared fluorescence probe 1 was successfully used to identify ESKAPE (Enterococcus faecium, Staphylococcus aureus, Klebsiella pneumoniae, Acinetobacter baumannii, Pseudomonas aeruginosa, and Enterobacter spp.) pathogens based on their NTR activities [27].

Bacterial probes, including Vanco-800CW and labeled antibodies targeting the immunodominant Staphylococcal antigen A (1D9-680), were positively applied to efficiently debride the infected tissue in animal models. These experiments re-emphasize the role of imaging in the surgical debridement of infected tissues [28].

Bacteria that have the ability to form biofilms cause infections that are difficult to treat and are resistant to antimicrobial treatment. Biofilms are a complex organization of bacterial colonies on implant devices and mammalian tissues that enable the survival of bacteria for long periods and make them impermeable to antibiotics. Sonobactericide is a process wherein microbubbles and/or droplets are exposed to ultrasound, which enables the effective killing of bacteria within biofilms [29].

Application of the d-[methyl-11C]methionine (d-[11C]Met) probe in mouse models was efficient in the detection of live bacterial infections from sterile inflammations based on PET scans [30].

The diagnosis of active pulmonary tuberculosis (PTB) caused by Mycobacterium tuberculosis is highly significant in preventing the spread of infection from one person to another. Radiological evidence utilizing X-ray imaging plays a key role in the diagnosis and prognosis of PTB [31].

Different radiological signs on CT scans could be used to identify infections caused by microbes, including bacteria, fungi, parasites, and viruses. Most bacterial infections of the lungs show consolidation and air bronchogram signs on CT scans. A silhouette sign on a CT scan is more common among patients with bacterial pneumonia. The tree-in-bud sign is frequently noticed among patients suffering from tuberculosis. The bulging fissure sign indicates a Klebsiella infection. Other radiological/CT features noticed during bacterial infections and cancers include inhomogeneous enhancement signs and cavitation, air-fluid level signs, and split-pleura signs [32]. The various diagnostic imaging approaches discussed above have been summarized in Table 1.

**Table 1:** Different radiological and imaging approaches to diagnose bacterial infections NTR: nitroreductase, CT: computed tomography, PET: positron emission tomography, ESKAPE: *Enterococcus faecium*, *Staphylococcus aureus*, *Klebsiella pneumoniae*, *Acinetobacter baumannii*, *Pseudomonas aeruginosa*, and *Enterobacter spp.*

Method	Application	Examples	Usefulness
Radiolabeling	Radiolabeled antibiotics, antimicrobial proteins/peptides, metabolites/ligands, bacteriophages, and nucleic acid degradation materials	*Pseudomonas aeruginosa* specific antibody (^125^Iodine), [68 Ga] Ga-NODAGA-PEG2-P2 that targets *Escherichia coli*	Diagnosis of bacterial infections
Multicolor fluorescence imaging device (FluoroPi)	Animal model/animal experiments	Animal models of microbial keratitis	FluoroPi utilizes fluorescent optical reporters/Smart probes to differentially identify Gram-positive and Gram-negative bacterial infections
Microbial metabolism	Animal model/animal experiments	lung infection models	Variations in the L-and D-amino acids were useful in predicting bacterial infections through imaging
	NTR activity	Near-infrared fluorescence probe 1	Used to differentiate between cancer-related fluid accumulations/inflammation from bacterial infections and identify ESKAPE pathogens
Probes	Maltodextrin-based probes	Imaging	Used to identify bacteria-mediated fluid accumulations
	Vanco-800 CW and labeled antibodies targeting the immunodominant Staphylococcal antigen A (1D9-680)	Animal models	Used to efficiently debride the infected tissue in animal models
	d-[methyl-11C] methionine (d-[11C] Met) probe	Animal models	Used to detect live bacterial infections from sterile inflammations based on PET scans
Bacteria-targeted fluorescence imaging	Near-infrared fluorescent tracer composed of the antibiotic vancomycin and the fluorophore IRDye800CW (vanco-800 CW)	Human subjects	To identify bacterial colonization on the implant devices and diagnose fracture-related infections
Imaging	X-rays and CT scans	Human subjects	Most bacterial infections of the lungs show consolidation and air bronchogram signs on CT scans. The bulging fissure sign indicates *Klebsiella* infection.

Fungal infections and imaging

Although CT scans can show radiological signs, it is extremely difficult to differentially diagnose bacterial, fungal, and parasitic infections. The CT images depict a halo sign; the air crescent sign indicates angioinvasive Aspergillus infection; and the air crescent or monod sign suggests fungal mycetoma. The finger-in-glove sign, reverse halo, bird's nest sign, and crazy-paving sign are common among fungal infections [32].

Invasive fungal infections (IFIs) have seen an exponential rise in the past decade, mostly owing to growth in the debilitated and immunocompromised population. Timely diagnosis of IFIs is extremely important to minimize morbidity and mortality. Despite the availability of established diagnostic modalities like microscopy, culture, serology, and molecular methods, the diagnosis of fungal infections remains complex owing to the difficulties in acquiring appropriate clinical specimens. Therefore, more feasible and reliable diagnostic methods, like using radiological evidence to identify fungal infections, have been under investigation over the past decade.

Chest X-rays are the most common imaging method used to detect the location of infections. However, the changes noticed in X-rays may not conclusively convey the etiological agent involved in the infection. Fungal infections of the lungs may present as parenchymal opacities, pleural effusions, and cavitated nodules in the X-ray images. However, these features are inconclusive about the fungal etiology. Therefore, CT imaging has been suggested as superior in the identification of IFIs. The observation of multiple bilateral nodules surrounded by ground glass opacities (the halo sign) was identified as a CT feature suggestive of invasive Aspergillosis. Additionally, bilateral consolidation of the lungs along with cavitation and nodules were evident in Candida infections. It was revealed that MRI and PET scans combined could be more efficient in the identification of lesions caused by fungi. These imaging modalities could aid disease prognosis and treatment responses. Besides, individuals with hematological malignancies are increasingly affected by IFIs that involve the central nervous system (CNS) and other organs of the body, like the liver, that can be efficiently diagnosed in the early stages of infection using PET/MRI [33].

CT imaging of pulmonary lesions among human immunodeficiency virus (HIV)-infected persons was assessed before and after anti-fungal treatment. The results of this study demonstrated that the CT scans were sufficient to evaluate the prognosis, as evidenced by the resolution of pulmonary lesions following treatment [34].

Despite CT imaging being a preferred diagnostic modality in diagnosing IFIs for two decades, the efficiency of CT scans has increased in recent times. Additionally, 18F-fluorodeoxyglucose (FDG) PET/CT is currently being applied to monitor treatment responses. It was suggested that the development of fungal-specific antibody imaging tracers could improve the accuracy of the diagnosis and prognosis of IFIs [35].

In a retrospective multi-center study that assessed CT imaging, it was observed that patients suffering from invasive Aspergillus and non-Aspergillus infection (IANA) demonstrated multiple nodules and fewer ground glass opacities. Additionally, this study’s results revealed that IFIs of the lungs show characteristic features like nodules, ground glass opacities, and consolidations on CT imaging [36].

The application of USG and CT scans among pediatric-age patients (7.16±4.23 years) with underlying hematological malignancies revealed that IFIs could be diagnosed using USG (61.9%) and CT (71.4%) images. Additionally, a combination of USG and CT images could have higher sensitivity (82.4%) and specificity (76.5%) in the diagnosis of IFIs [37].

A previous study observed that the radiological imaging features of fungal diseases are indistinguishable from cancerous lesions. Such similarities could lead to the misdiagnosis of common fungal infections as cancers/tumors and contribute to a delay in the initiation of antifungal treatment [38].

The major limitation of fungal infections is that they cause lesions that evolve gradually over a long period of time. Therefore, imaging techniques may fail to demonstrate the changes at the early stages of infection.

Parasitic infections and imaging

A majority of parasitic infections are localized in the intestines. However, some parasitic infections lead to the movement and localization of the parasite within internal organs like the liver, brain, and other organs of the body. Parasitic infections on CT imaging appear as meniscus, cumbo, and water lily signs in cases of echinococcal infection and burrow signs revealing paragonimiasis [32].

Imaging studies could be increasingly helpful in the diagnosis of parasitic infections when the clinical symptoms are inconclusive, especially when the parasite invades the tissues, organs, and muscles [39]. Since parasitic diseases demonstrate similar imaging features as other infections and cancerous conditions, differential diagnosis assumes increased significance. The application of advanced imaging techniques with high contrast, like MRI, PET-CT, and nuclear medicine techniques, could improve specific diagnoses of parasitic diseases [40].

Because parasites localize deep inside the body, the collection of specimens and extraction of the parasites become increasingly difficult. Therefore, non-invasive methods like radiological imaging could be beneficial in the identification of the parasite location [41].

Parasitic diseases such as amoebiasis, cysticercosis, malaria, toxoplasmosis, cystic echinococcosis, filariasis, and others produce lesions in the brain, liver, and other organs that could be visualized through CT, MRI, and other advanced imaging modalities like fluid attenuation inversion recovery (FLAIR), diffusion MRI, perfusion MRI, MRI spectroscopy, and three-dimensional MRI sequences, including Fast Imaging Employing Steady-state Acquisition (FIESTA) and Spoiled Gradient Recalled Echo (SPGR) techniques [42,43].

Radiologists and imaging specialists frequently encounter lesions that suggest parasitic infections, like the amoebic liver abscess caused by Entamoeba histolytica, while performing USG, CT, and MRI. The location of the lesions (near the capsule) and the type of lesion (enhancing the thick wall with perilesional edema) could assist in the accurate diagnosis of extra-intestinal amoebiasis. Additionally, specific imaging changes could be used to identify other parasitic infections like toxoplasmosis of the brain, Chagas disease/American trypanosomiasis (Trypanosoma cruzi), leishmaniasis, echinococcosis, cysticercosis, Ascariasis, strongyloidiasis, and schistosomiasis, among others [40].

Viral infections and imaging

Radiological imaging during viral infections is particularly useful in the assessment of the involvement of the lungs by respiratory viral infections like the SARS-CoV-2 and Influenza virus, among others. Especially during the initial phases of the SARS-CoV-2 pandemic, when there was no laboratory method for the diagnosis of infection, CT imaging was used to stage the disease and manage the patients suffering from Coronavirus disease 2019 (COVID-19). Chest CT-based Viral Pneumonia Imaging Reporting and Data System (VP-RADS) was suggested by radiologists to stage COVID-19. The VP-RADS uses CT images showing ground-glass opacities (GGOs) along with the history of exposure and clinical and laboratory findings to provisionally diagnose SARS-CoV-2 infection. According to the VP-RADS, patients were classified into four categories (categories 1-4), wherein category four patients were considered to be potentially infected by SARS-CoV-2 [44].

Pneumonia is another clinical condition that requires radiological imaging to assess the extent of infection during patient management. Additionally, it is essential to differentiate between viral and bacterial pneumonia based on imaging features to minimize the unwarranted use of antibiotics [45].

CT features of pneumonia-causing viruses other than SARS-CoV-2 were found to be marginally different. These differences could be used to diagnose viral cases of pneumonia caused by Adenovirus (multifocal consolidations and GGOs), Rhinovirus (multifocal GGOs with and without consolidation), SARS-CoV-1 and Middle East Respiratory Syndrome (MERS) virus (multifocal consolidation with GGOs with peripheral predominance), Influenza virus (multifocal consolidation with or without GGOs and central/peripheral distribution), human Parainfluenza virus (multifocal consolidation, GGOs, and centrilobular nodules), respiratory syncytial virus (RSV) (multifocal consolidation and centrilobular nodules with an airway centric pattern), human metapneumovirus (centrilobular nodules with bronchiolitis patterns), and cytomegalovirus (diffuse GGOs with or without multiple ill-defined tiny nodules) [46].

Other viruses that can infect lungs, like Herpes virus (multifocal areas of segmental or sub-segmental GGO are observed; pleural effusion is frequent), varicella zoster virus (VZV) (well-defined nodules (1-10 mm) with a halo of GGOs and calcified lesions), and Epstein-Barr virus (EBV) (lymphadenopathies and less frequently interstitial infiltrates with diffuse GGOs and consolidations) were also differentiated based on the characteristic CT findings [47,48].

Recently, a transfer learning-based multi-class convolutional neural network model was suggested to differentiate COVID-19 pneumonia from other viral cases of pneumonia. This model uses chest X-rays to provide information regarding the type of infection based on the structural changes in the lungs [49].

The application of machine learning, deep learning, and convolutional neural network models to diagnose and differentiate viral pneumonia was recently reported. These models have been applied to chest X-ray images that are easily available and affordable to patients. Based on these CNN models, the patients could be classified into various categories, like negative for pneumonia, typical pneumonia, indeterminate type, and atypical pneumonia [50].

Different viruses have the ability to infect the CNS and result in clinical conditions such as meningitis, encephalitis, encephalomyelitis, and encephalomyeloradiculitis. Viral infections of the CNS result in changes in the brain that are non-specific and that cannot conclusively suggest viral etiology. Therefore, radiological and imaging evidence must be correlated with culture, serology, and pathological features [51].

Despite the limitations of radiological and imaging methods to accurately diagnose viral liver diseases like hepatitis, it was suggested that CT and MRI findings like hepatomegaly, periportal lymphadenopathy, periportal edema, ascites, and a thickened and edematous gall bladder wall could indicate acute or chronic viral hepatitis [52].

## Conclusions

Radiological investigations have become increasingly available and efficiently applied in the diagnosis and management of infectious diseases. However, it is extremely difficult to diagnose microbial infections based on anatomical abnormalities. Additionally, radiological images fail to accurately predict and differentiate between microbial infection-related anomalies, sterile inflammations, and cancer-related changes. Nuclear medicine advances that utilize radiolabeled probes have been successfully employed in the diagnosis and management of microbial infections in recent times. Further, machine learning and other computational methodologies could be applied to aid the diagnosis of infectious diseases based on radiological evidence. Increased research in this direction could, in the future, enhance the usefulness of radiological evidence in the diagnosis and management of microbial infections.
